# The clinical relevance of serum antinuclear antibodies in Japanese patients with systemic sclerosis

**DOI:** 10.1111/j.1365-2133.2007.08392.x

**Published:** 2008-03-01

**Authors:** Y Hamaguchi, M Hasegawa, M Fujimoto, T Matsushita, K Komura, K Kaji, M Kondo, C Nishijima, I Hayakawa, F Ogawa, M Kuwana, K Takehara, S Sato

**Affiliations:** Department of Dermatology, Kanazawa University Graduate School of Medical Science Kanazawa, Ishikawa, Japan; * Department of Dermatology, Nagasaki University Graduate School of Biomedical Sciences 1-7-1 Sakamoto, Nagasaki 852-8501, Japan; † Department of Internal Medicine, Keio University School of Medicine Tokyo, Japan

**Keywords:** antinuclear antibodies, clinical features, survival, systemic sclerosis

## Abstract

**Background:**

Systemic sclerosis (SSc) is a connective tissue disorder with excessive fibrosis of the skin and various internal organs. Although SSc is a heterogeneous disease, it has been reported that the particular antinuclear antibodies (ANA) are often indicative of clinical features, disease course and overall severity.

**Objective:**

To clarify the association of clinical and prognostic features with serum ANA in Japanese patients with SSc.

**Methods:**

We studied 203 Japanese patients diagnosed with SSc, who visited our hospital during the period 1983–2005. Six SSc-related ANA were identified using indirect immunofluorescence, double immunodiffusion and immunoprecipitation assays.

**Results:**

Patients with SSc were classified into six ANA-based subgroups and a group without ANA. As expected, antitopoisomerase I antibody (Ab, *n* = 64), anti-RNA polymerases (RNAP) Ab (*n* = 12) and anti-U3 RNP Ab (*n* = 5) were associated with diffuse cutaneous SSc, whereas anticentromere Ab (ACA, *n* = 75), anti-Th/To Ab (*n* = 7) and anti-U1 RNP Ab (*n* = 10) were frequently detected in patients with limited cutaneous SSc. Clinical features of the ANA-negative group (*n* = 10) were heterogeneous. Consistent with previous findings in Caucasian and/or black African patients, antitopoisomerase I Ab was associated with the involvement of vascular and pulmonary fibrosis, leading to decreased survival rate. However, no patients with anti-RNAP Ab developed renal crisis and the frequency of isolated pulmonary hypertension in patients with ACA, anti-Th/To Ab or anti-U3 RNP Ab was similar to that in other ANA-based subgroups.

**Conclusion:**

These results indicate that the clinical relevance of SSc-related ANA in Japanese patients differs in some aspects from that in Caucasian and/or black African patients.

Systemic sclerosis (SSc) is a connective tissue disorder characterized by microvascular damage and excessive fibrosis of the skin and various internal organs. There are variations of clinical expression in patients with SSc, ranging from limited cutaneous SSc (lSSc), in which skin thickening is relatively restricted to the fingers and hands, and with less serious internal organ involvement, to diffuse cutaneous SSc (dSSc), in which skin lesions are extensive and often rapidly developing, with earlier and more serious complications.^1^ One representative feature of the immunological abnormalities in patients with SSc is the presence of antinuclear antibodies (ANA). More than 90% of patients with SSc are positive for ANA, and these ANA react to various intracellular components.^2^ Several major findings with regard to ANA have been reported previously in patients with SSc. Production of specific ANA is exclusive and one patient rarely has two or more SSc-related specific ANA.^3^ If once ANA have appeared, usually they do not change into other specific ANA or disappear during the disease course.^4^ Although the role of ANA in the pathogenesis of SSc remains unknown and they may not be directly involved, particular ANA are often indicative of clinical features, disease course and overall severity. Therefore, information on ANA is a valuable help in diagnosing and evaluating the prognosis of an individual patient with SSc.

Anti-DNA topoisomerase I antibody (anti-topo I; formerly termed anti-Scl-70)^5^ and anticentromere antibody (ACA)^6^ are two representative ANA found in SSc. Other serum ANA associated with SSc include anti-U1 ribonucleoprotein (RNP),^7^ anti-RNA polymerases I, II and III (anti-RNAP),^8^,^9^ anti-Th/To^10^ and anti-U3 RNP (fibrillarin)^11^ antibodies. It has been reported that there are several clinical associations with these ANA. ACA^12^–^14^ and anti-Th/To^15^,^16^ are associated with lSSc and with a low frequency of severe internal organ involvement, although some patients with these ANA develop isolated pulmonary arterial hypertension (PAH). In contrast, anti-topo I is generally associated with dSSc and a high frequency of pulmonary fibrosis.^17^,^18^ Anti-RNAP is frequently detected in patients with dSSc and is associated with a higher proportion of male patients, the onset of disease at a greater age and a high frequency of renal disease.^8^,^9^ Anti-U1 RNP is often found in overlap syndrome, especially mixed connective tissue disease, and is associated with isolated PAH and arthritis.^19^ Anti-U3 RNP is generally detected in patients with dSSc with a low frequency of joint and pulmonary involvement or with isolated PAH, skeletal muscle involvement and early disease onset.^20^ Approximately 5–10% of patients with SSc are likely to have no serum ANA. The detailed clinical presentation in this subgroup remains unclear.

It is well known that ethnic and genetic background affect frequencies of ANA and clinical features in patients with SSc.^21^ Most reports have described demographic and clinical features in Caucasian and/or black African patients with SSc and relatively small studies have been performed on Japanese patients with SSc. In this study, we attempted to identify a correlation between SSc-related ANA and clinical features and prognosis in 203 Japanese patients with SSc.

## Patients and methods

### Patients and serum samples

We analysed serum samples from 203 Japanese patients with SSc (173 women and 30 men). All patients fulfilled the criteria for classification of definite SSc proposed by the American College of Rheumatology.^22^ The mean age of the patients was 46 ± 15 years and the disease duration was 6·6 ± 8·3 years. The disease duration was calculated from the onset of the first clinical event that was a clear manifestation of SSc. At their first visit, 12 patients had been treated with low-dose corticosteroid (prednisolone 5–20 mg daily) and 19 patients with d-penicillamine (100–300 mg daily). None of the patients with SSc had received other immunosuppressive therapies. The patients were grouped according to the classification system proposed by LeRoy *et al.*:^1^ 112 patients (104 women and eight men) had lSSc and 91 patients (69 women and 22 men) had dSSc. Patients with SSc in overlap syndrome, which is mostly characterized by SSc with systemic lupus erythematosus or polymyositis, were excluded from this study.

At the most recent observation point, 185 patients were still alive. The mean follow-up period including 18 deceased patients was 7·1 ± 4·3 years. Seventy-seven patients received corticosteroid (prednisolone 5–30 mg daily) and seven received low-dose d-penicillamine (100–300 mg daily) throughout the follow-up period. One patient received steroid pulse therapy, followed by oral prednisolone 40 mg daily for subacute deterioration of interstitial pneumonitis during the follow-up period. Fresh venous blood samples were centrifuged shortly after clot formation. All samples were stored at −70 °C before use.

The protocol was approved by the Kanazawa University School of Medical Science and Kanazawa University Hospital, and informed consent was obtained from all study subjects.

### Antinuclear antibody analysis

**Fig 1:**
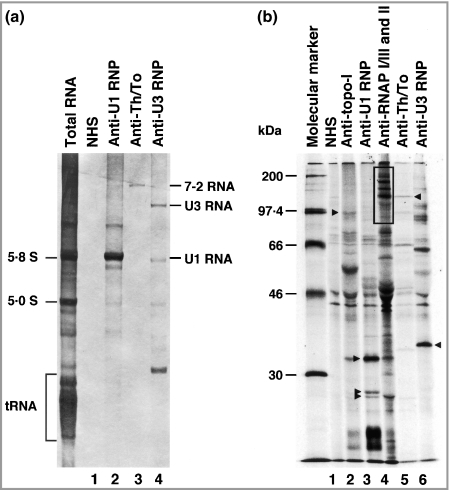
Immunoprecipitation assay of autoantibodies related to systemic sclerosis (SSc). (a) Immunoprecipitation of U1 ribonucleoprotein (RNP), Th/To and U3 RNP by sera. K562 cell extracts were immunoprecipitated with sera and RNA was extracted, run on 8% urea–polyacrylamide gel electrophoresis (PAGE), visualized by silver staining. Total RNA, with the 7·0, 5·8 and 5·0 S small ribosomal RNAs and the tRNA region indicated; lane 1, normal human serum (NHS); lane 2, anti-U1 RNP-positive sera; lane 3, anti-Th/To-positive sera; lane 4, anti-U3 RNP-positive sera. (b) Immunoprecipitation of ^35^S-methionine-labelled K562 cell extracts was performed on sera from patients with SSc (lanes 2–6) and NHS, separated on 10% SDS-PAGE, and analysed by autoradiography. Molecular weight marker includes protein bands corresponding to 200, 97·4, 66, 46 and 30 kDa. Topoisomerase-I (topo-I, lane 2), U1 RNP (lane 3), RNA polymerase I/III, and II (RNAP I/III and II, lane 4), Th/To (lane 5) and U3 RNP (lane 6) proteins are shown by arrowheads or indicated region.

A total of 203 serum samples from Japanese patients with SSc were analysed for ANA. Indirect immunofluorescence tests were performed using slides of monolayer HEp-2 cells (Medical & Biological Laboratories, Nagoya, Japan) as substrate.^8^ Ouchterlony double immunodiffusion tests and immunoprecipitation assays using nonradiolabelled and ^35^S-methionine-labelled K562 cell extracts^23^,^24^ were also carried out (Fig. 1a,b). Six well-known SSc-related ANA were defined in this study as follows. ACA was considered positive if serum diluted at 1 : 40 produced a characteristic staining pattern on HEp-2 cells as well as on commercially prepared HeLa cell chromosomal spreads (Medical & Biological Laboratories).^6^ Anti-U1 RNP,^25^ anti-Th/To^10^ and anti-U3 RNP^20^ antibodies were detected by immunoprecipitation using nonradiolabelled K562 cell extracts. Immunoprecipitated RNA was detected in 8% urea–polyacrylamide gel electrophoresis (PAGE) from a cell extract obtained from 3 × 10^6^ K562 cells by phenol/chloroform, visualized by silver staining. Anti-RNAP^8^ was detected by immunoprecipitation of proteins detected in sodium dodecyl sulphate (SDS)-PAGE from a cell extract obtained from 1 × 10^6^ K562 cells radiolabelled with 50 μCi of ^35^S-methionine. Anti-topo I was considered positive if serum samples produced precipitin lines with immunological identity to reference sera on double immunodiffusion, or if it immunoprecipitated a protein with a molecular weight of ∼100 kDa from ^35^S-methionine-labelled K562 cell extracts. We defined sera without the presence of any characteristic staining on HEp-2 cells using sera diluted to 1 : 40 as negative for ANA.

### Clinical assessment

At their first visit, complete medical histories were obtained, and physical examinations and laboratory tests were carried out for most of the patients, with limited evaluations during follow-up visits. Serum samples and clinical laboratory data were obtained at the same time. Skin thickness was scored according to the modified Rodnan scoring technique.^26^ Briefly, the 17 anatomic areas were rated as 0 (normal skin), 1+ (mild but definite skin thickening), 2+ (moderate skin thickening) and 3+ (severe skin thickening), and the modified Rodnan total skin thickness score (TSS) was derived by summation of the scores from all 17 areas (range 0–51). Organ system involvement was defined as described previously:^27^,^28^ lung = bibasilar fibrosis on chest radiography and high-resolution computed tomography; isolated PAH = clinical evidence of pulmonary hypertension and increased systolic pulmonary arterial pressure (> 35 mmHg) by echocardiography, in the absence of severe pulmonary interstitial fibrosis; oesophagus = hypomotility shown by barium radiography; joint = inflammatory polyarthralgias or arthritis; heart = pericarditis, congestive heart failure or arrythmias requiring treatment; kidney = malignant hypertension and rapidly progressive renal failure without any other explanation; and muscle = proximal muscle weakness and elevated serum creatine kinase concentration. Pulmonary function tests, including vital capacity (VC) and diffusion capacity for carbon monoxide (DLco), were also performed. When the DLco and VC were < 75% and < 80%, respectively, of the predicted normal values, they were considered abnormal. Two comparisons were conducted to clarify the associations of SSc-related ANA with their clinical features. Firstly, we compared the frequencies of SSc-related demographic and clinical features in each SSc-related ANA-based subgroup with the total group of 203 patients with SSc to make the properties of each SSc-related ANA clear in the total population of SSc patients. To clarify the differences in clinical features among the SSc-related ANA-based subgroups, mutual comparisons were then performed among six SSc-related ANA-based subgroups and the ANA-negative SSc patient group.

### Statistical analysis

Statistical analysis was performed using Fisher's exact probability test for comparison of frequencies and the Mann–Whitney *U* test for comparison of values. The Bonferroni adjustment was used for multiple comparisons. Cumulative survival rates were calculated according to life-table methods, and comparisons were made using log-rank tests. *P* < 0·05 was considered statistically significant. All data are shown as mean ± SD.

## Results

### Detection and frequencies of antinuclear antibodies in Japanese patients with SSc

In sera from 203 Japanese patients with SSc, ACA was present in 77 (38%), anti-topo I in 67 (33%), anti-RNAP in 12 (6%), anti-U1 RNP in 11 (5%), anti-Th/To in seven (3%), anti-U3 RNP in five (2%), and no presence of ANA in 10 patients (5%). Seventeen patients (8%) had ANA which were not identified by analysis. Three patients had two kinds of the SSc-related ANA: two had anti-ACA in addition to anti-topo I and one had anti-topo I plus anti-U1 RNP. Thus, at least one kind of SSc-related ANA was present in the sera of 176 patients. In patients with anti-topo I, 14 were also positive for anti-SS-A and two for both anti-SS-A and -SS-B. Patients were then grouped according to ANA specificity with the following exception: the three patients with two kinds of SSc-related ANA were excluded from each ANA-based group (Table 1).

**Table 1:** Clinical and laboratory characteristics of 203 Japanese patients with systemic sclerosis (SSc), classified according to the presence of six SSc-related antinuclear antibodies (ANA) or absence of ANA^a^

	Total (*n* = 203)	Anti-topo I (*n* = 64)	Anti-ACA (*n* = 75)	Anti-U1 RNP (*n* = 10)	Anti-RNAP (*n* = 12)	Anti-Th/To (*n* = 7)	Anti-U3 RNP (*n* = 5)	Negative (*n* = 10)
Sex, female : male(% female)	173 : 30 (85)	52 : 12 (81)	71 : 4 (95)	7 : 3 (70)	7 : 5 (58)	5 : 2 (71)	5 : 0 (100)	10 : 0 (100)
Age at onset (years),mean ± SD	46 ± 15	43 ± 18	47 ± 14	42 ± 12	53 ± 16	53 ± 2	45 ± 19	49 ± 7
Duration (years),mean ± SD	6·6 ± 8·3	5·1 ± 6·6	9·7 ± 9·9	8·8 ± 9·8	2·6 ± 2·8	2·0 ± 2·2	1·9 ± 1·8	2·2 ± 2·4
Disease subset								
dSSc : lSSc (% of dSSc)	91 : 112 (45)	53 : 11 (83)**	4 : 71 (5)**	2 : 8 (20)	12 : 0 (100)**	1 : 6 (14)	4 : 1 (80)	3 : 7 (30)
Modified RodnanTSS	10·8 ± 9·4	16·1 ± 10·1*	5·8 ± 5·7**	5·4 ± 5·4	20·7 ± 10·6**	7·1 ± 9·3	16·6 ± 4·3	7·1 ± 6·7
Clinical features								
Pitting scars	35	58	17	50	25	29	80	10
Contracture of phalanges	44	50	23	30	83	43	60	30
Diffuse pigmentation	43	55	21	50	92*	29	60	30
Organ involvement								
Lung								
Pulmonary fibrosis	44	84**	7**	30	17	29	0	10
Isolated PAH	14	17	7	20	8	14	0	10
Decreased %VC	22	44*	4*	30	17	29	0	10
Decreased %DLco	61	78	47	60	58	29	20	70
Oesophagus	65	68	57	80	60	29	100	60
Heart	16	20	12	10	17	14	20	10
Kidney	1	5	0	0	0	0	0	0
Joint	29	31	23	50	50	14	40	20
Muscle	10	13	7	0	17	14	20	20
Laboratory findings, mean ± SD								
ESR (mm h^−1^)	22 ± 22	24 ± 21	20 ± 18	40 ± 40*	17 ± 17	18 ± 10	15 ± 8	15 ± 11
CRP (mg dL^−1^)	0·3 ± 0·7	0·6 ± 1·1	0·2 ± 0·4	0·5 ± 1·0	0·3 ± 0·3	0·3 ± 0·2	0·1 ± 0·1	0·3 ± 0·5
IgG (mg dL^−1^)	1685 ± 590	1735 ± 491	1509 ± 395	2770 ± 1170**	1361 ± 457	1654 ± 448	1957 ± 941	1582 ± 420

a Unless noted otherwise, values are percentages. Three patients with two SSc-related antinuclear antibodies were excluded from each ANA-based group. Anti-topo I, anti-topoisomerase I; ACA, anticentromere; anti-U1 RNP, anti-U1 ribonucleoprotein; anti-RNAP, anti-RNA polymerase I, II and III; dSSc, diffuse cutaneous SSc; lSSc, limited cutaneous SSc; TSS, total skin thickness score; PAH, pulmonary arterial hypertension; VC, vital capacity; DLco, diffusing capacity for carbon monoxide; ESR, erythrocyte sedimentation rate; CRP, C-reactive protein.

* *P* < 0·05 vs. total SSc patients;

** *P* < 0·01 vs. total SSc patients.

### Demographic features

As shown in Table 1, 95% of the ACA-positive patients were female. This proportion was significantly higher than in anti-RNAP-positive patients (58%, *P* < 0·05). Patients with ACA or anti-U1-RNP showed a tendency for longer disease duration (8·8–9·7 years). In contrast, patients with anti-RNAP, anti-Th/To or anti-U3 RNP, and ANA-negative patients exhibited shorter disease duration (1·9–2·6 years). Patients with anti-topo I had an intermediate disease duration (5·1 years). ACA-positive patients had a significantly prolonged mean disease duration (9·7 ± 9·9 years) compared with anti-U3 RNP-positive (1·9 ± 1·8 years) and ANA-negative patients (2·2 ± 2·4 years) (*P* < 0·01).

### Disease classification

When patients with SSc were classified into the two disease subsets, the frequency of dSSc was significantly higher in patients with anti-topo I (83%, *P* < 0·01) or anti-RNAP (100%, *P* < 0·01) compared with the total patients with SSc (45%, Table 1). In contrast, the percentage of patients with dSSc was significantly lower among the patients with ACA (5%) than in the total SSc patient group (*P* < 0·01). Patients with anti-U1 RNP and those with anti-Th/To tended to have lSSc (80% and 86%, respectively), while 80% of anti-U3 RNP-positive patients had dSSc. The frequency of dSSc was 30% in ANA-negative patients. Thus, in general, anti-topo I, anti-RNAP and anti-U3 RNP were associated with dSSc, while ACA-, anti-U1 RNP- and anti-Th/To-positive patients exhibited lSSc.

### Modified Rodnan total skin thickness score

As skin thickening serves as an effective clinical tool for assessment of disease severity,^26^ direct correlation of each SSc-related ANA with the modified Rodnan TSS was assessed (Table 1). The modified Rodnan TSS of patients with anti-topo I (16·1 ± 10·1) or anti-RNAP (20·7 ± 10·6) was significantly higher than that of the total 203 patients with SSc (10·8 ± 9·4, *P* < 0·05 and *P* < 0·01, respectively). Patients with anti-U3 RNP had a tendency to have higher modified Rodnan TSS (16·6 ± 4·3). In contrast, the modified Rodnan TSS of ACA-positive patients (5·8 ± 5·7) was significantly lower than that of the total SSc population (*P* < 0·05). Patients with anti-U1 RNP, those with anti-Th/To and those without ANA showed lower TSS (5·4 ± 5·4, 7·1 ± 9·3 and 7·1 ± 6·7, respectively). These findings are generally in accordance with the findings that most of the patients with anti-topo I, anti-RNAP or anti-U3 RNP were classified as dSSc while patients with ACA, anti-U1 RNP or anti-Th/To were generally grouped into lSSc.

### Clinical assessment

We compared the frequencies of SSc-related organ involvement in each SSc-related ANA-based subgroup (Table 1). Pitting scars were seen more frequently in patients with anti-topo I, anti-U1 RNP or anti-U3 RNP than in patients with ACA, anti-RNAP or anti-Th/To and those without ANA, although the difference did not reach statistical significance. Contracture of phalanges was seen frequently in all subgroups, although it was relatively higher in patients with anti-RNAP and lower in patients with ACA or anti-U1 RNP and those without ANA. Diffuse pigmentation was found more frequently in patients with anti-RNAP (92%) than in the total patients (43%, *P* < 0·05). Patients with anti-topo I or anti-U3 RNP tended to have this symptom, but it was infrequently found in ACA-positive, Th/To-positive and ANA-negative patients. These findings suggest that contracture of phalanges or diffuse pigmentation is generally related to the extent of skin sclerosis. However, the frequency of pitting scars was not necessarily associated with the severity of skin sclerosis, as the scars were infrequent in anti-RNAP-positive patients.

Most organ system involvement and laboratory findings are primarily associated with SSc-related ANA, rather than the disease subsets.^29^ Pulmonary fibrosis (84%, *P* < 0·01 vs. total patients) and decreased %VC (44%, *P* < 0·05 vs. total patients) were most common in patients with anti-topo I and most infrequent in patients with ACA (7%, *P* < 0·01 and 4%, *P* < 0·05 vs. total patients, respectively, Table 1), as previously shown.^27^ Decreased %DLco was commonly seen in patients with SSc, except for patients with anti-Th/To (29%) or anti-U3 RNP (20%). The frequency of isolated PAH was between 0 and 20% in all subgroups and no significant difference was found. Upper gastrointestinal involvement, which is generally represented by oesophageal dysfunction, occurred in more than 50% of patients in all subgroups, except for patients with anti-Th/To (29%). Severe heart and muscle involvement was infrequent and occurred in 20% or less in all subgroups without any significant association with specific ANA. Only three patients experienced renal crisis and all of them had anti-topo I.

In laboratory findings, patients with anti-U1 RNP showed a significantly higher erythrocyte sedimentation rate and increased serum IgG levels than that in the total patient group (*P* < 0·05 and *P* < 0·01, respectively, Table 1).

### Comparison between anti-topo I- and anti-RNAP-positive patients with dSSc

Although the clinical significance of ANA is especially important in patients with dSSc, the features found in patients with anti-topo I or anti-RNAP may be due to the increased dSSc ratio rather than to the specific ANA. Therefore, we compared clinical and laboratory features in patients with dSSc with anti-topo I or anti-RNAP (Table 2). Patients with anti-RNAP showed a tendency towards short disease duration compared with patients with anti-topo I. The finding that modified Rodnan TSS was similar between the two groups suggests that patients with anti-RNAP show more rapid progression of skin sclerosis than patients with anti-topo I. Patients with anti-RNAP had significantly less frequent pitting scars (*P* < 0·05) and more frequent diffuse pigmentation (*P* < 0·05) compared with those with anti-topo I. The frequencies of pulmonary fibrosis and decreased %VC were significantly higher in patients with anti-topo I than in those with anti-RNAP (*P* < 0·01 and *P* < 0·05, respectively). Serum IgG concentrations in patients with anti-topo I were significantly higher than those in patients with anti-RNAP (*P* < 0·05). Thus, patients with anti-topo I or anti-RNAP showed some different features even within the dSSc subgroup.

**Table 2:** Clinical and laboratory characteristics of 65 Japanese patients with the diffuse form of cutaneous systemic sclerosis with anti-topoisomerase I (anti-topo I) or anti-RNA polymerase I, II and III (anti-RNAP) antibodies^a^

	Anti-topo I (*n* = 53)	Anti-RNAP (*n* = 12)
Age at onset (years),mean ± SD	43 ± 19	53 ± 16
Sex, female: male(% of female)	42 : 11 (79)	7 : 5 (58)
Duration (years),mean ± SD	5·2 ± 6·7	2·6 ± 2·8
Modified Rodnan TSS,mean ± SD	18·5 ± 9·4	20·7 ± 10·6
Clinical features (%)		
Pitting scars	62*	25
Contracture of phalanges	68	83
Diffuse pigmentation	62*	92
Organ involvement (%)		
Lung		
Pulmonary fibrosis	85**	17
Isolated PAH	21	8
Decreased %VC	47*	17
Decreased %DLco	77	58
Oesophagus	71	60
Heart	21	17
Kidney	6	0
Joint	34	50
Muscle	13	17
Laboratory findings (%), mean ± SD		
ESR (mm h^−1^)	25 ± 23	17 ± 17
CRP (mg mL^−1^)	0·6 ± 1·1	0·3 ± 0·3
IgG (mg mL^−1^)	1755 ± 528*	1361 ± 457

a Unless noted otherwise, values are percentages as shown in Table 1. TSS, total skin thickness score; PAH, pulmonary arterial hypertension; VC, vital capacity; DLco, diffusing capacity for carbon monoxide; ESR, erythrocyte sedimentation rate; CRP, C-reactive protein.

* *P* < 0·05 vs. patients with anti-RNAP;

** *P* < 0·01 vs. patients with anti-RNAP.

### Survival rates

**Fig 2:**
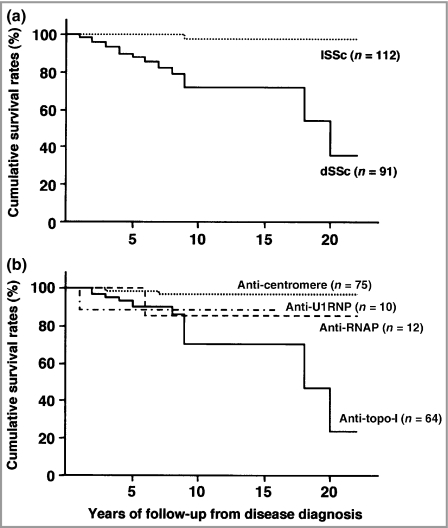
Cumulative survival rates from the time of diagnosis: (a) 203 Japanese patients with diffuse cutaneous systemic sclerosis (dSSc) and limited cutaneous SSc (lSSc); (b)161 Japanese patients with SSc with serum anticentromere, anti-U1 ribonucleoproteins (RNP), anti-DNA topoisomerase I (topo-I) and anti-RNA polymerase I, II and III (anti-RNAP) antibodies. Cumulative survival rates were compared using log-rank tests.

Overall cumulative rate of survival from the time of diagnosis of SSc in the 203 patients with SSc was 97% at 5 years and 90% at 10 years. As it has been reported that prognosis in patients with SSc is associated with the SSc disease subsets, we assessed the survival rates between the disease subsets. The cumulative survival rate was significantly decreased in the 91 patients with dSSc compared with the 112 patients with lSSc after 10 years [92% vs. 100% at 5 years (not significant) and 77% vs. 98% at 10 years (*P* < 0·01); Fig. 2a]. We then compared the survival rate among four ANA-based subgroups (Fig. 2b). The survival rate for patients with anti-topo I was 91% at 5 years and 70% at 10 years. This was the worst survival rate and was significantly lower than the rate in the ACA-positive subgroup (*P* < 0·01). No significant differences were found between ACA-, anti-U1 RNP- and anti-RNAP-positive patients. Thus, only the patient group with anti-topo I showed a decreased survival rate during the follow-up period.

### Cause of death

Table 3 shows the number of patients with SSc, classified by ANA-based subgroup, who died and the causes of their death. Sixteen of the 203 patients with SSc died during the follow-up period. Of 11 patients with anti-topo I, six died of respiratory failure or infectious pneumonia due to pulmonary fibrosis and three died of renal involvement which was typical hypertensive crisis. One anti-topo I-positive patient died of a malignancy and one of massive alveolar haemorrhage, unrelated to SSc. Only two of the 75 patients with ACA died; one of them died of right-sided cardiac failure due to isolated PAH. Of the 12 anti-RNAP-positive patients, one died of cardiac infarction. One ANA-negative patient died of a continuous bleeding in both the upper and lower gastrointestinal tract, where the upper lesion was characterized by gastric antral vascular ectasia. One patient with anti-U1 RNP died from a malignancy unrelated to SSc. None of the patients with anti-Th/To or anti-U3 RNP died during the follow-up period. Thus, the major cause of death in patients with SSc was associated with SSc-related internal organ involvement.

**Table 3:** Frequency and cause of death in Japanese patients with systemic sclerosis (SSc), classified according to the presence of six SSc-related antinuclear antibodies (ANA) or absence of ANA

Antinuclear antibody	No. of patients	Cause of death (*n*)
Anti-DNAtopoisomerase I	11	Pulmonary fibrosis (6),kidney involvement (3),malignancy (1),alveolar haemorrhage (1)
Anticentromere	2	Isolated pulmonary arterialhypertension (1), unknown (1)
Anti-U1 RNP	1	Malignancy (1)
Anti-RNApolymeraseI, II, and III	1	Cardiac infarction (1)
Anti-Th/To	0	–
Anti-U3 RNP	0	–
ANA-negative	1	Gastrointestinal involvement (1)

## Discussion

In this study of Japanese patients, we compared clinical features and prognosis in SSc subgroups with each SSc-related ANA. Consistent with previous reports, we found strong associations between SSc-related ANA and demographic and clinical features as follows: anti-topo I with dSSc and pulmonary fibrosis; ACA with female patients, lSSc and longer disease duration; anti-RNAP with dSSc; anti-Th/To with lSSc; and anti-U3 RNP with dSSc (Table 1 and Refs 29 and 30). However, our patients generally showed less frequent involvement of isolated PAH or renal crisis compared with previous reports in other countries. As a result, patients with ACA or anti-RNAP showed a remarkably favourable prognosis as did those with anti-U1 RNP. The survival rate was lowest in patients with anti-topo I; this subgroup frequently developed pulmonary fibrosis, as has been found in other ethnic groups.

The frequency of ANA in this study was 95%, consistent with previous studies.^30^–^32^ Anti-topo I was positive in 33% of 203 patients with SSc. This frequency was approximately equal to previous studies of Japanese patients and relatively higher than in Caucasian or black African patients.^29^–^32^ Our patients with SSc had a relatively higher percentage of ACA (38%) compared with 16–22% of patients in other studies.^29^–^32^ SSc in overlap syndrome is more common in Japanese compared with Caucasian or black African patients.^30^ However, only 5% of patients with SSc in our study had anti-U1 RNP. This may be explained by the fact that patients with SSc in overlap were excluded from this analysis, and this antibody significantly associates with overlap syndrome.^30^ In contrast, frequencies of anti-Th/To, anti-U3 RNP and anti-RNAP were comparable with those previously reported in Japanese and non-Japanese patients with SSc.^15^,^20^,^29^–^32^ Thus, frequencies of each SSc-related ANA were generally comparable with those previously described for Japanese patients with SSc.

Significant association of ANA with skin involvement has been described.^29^,^30^,^33^ Generally, patients with anti-topo I, anti-RNAP or anti-U3 RNP tend to present with dSSc and patients with ACA, anti-U1 RNP or anti-Th/To frequently have lSSc. In our study, 83% of anti-topo I-, 100% of anti-RNAP- and 80% of anti-U3 RNP-positive patients had dSSc, while 95% of ACA-, 80% of anti-U1 RNP- and 86% of anti-Th/To-positive patients had lSSc (Table 1). Our results were comparable with those previously seen in Japanese and non-Japanese patients with SSc.^29^,^30^ Thus, the development of skin sclerosis is strongly associated with SSc-related ANA and does not appear to be affected by ethnicity.

Anti-topo I and anti-RNAP are the representative SSc-specific antibodies whose clinical presentation is characterized by dSSc. Both show a diffuse type of skin thickening, but vasculopathy and pulmonary fibrosis are more frequently seen in patients with anti-topo I.^29^,^30^ We confirmed previous findings that patients with anti-topo I had higher frequencies of pitting scars and pulmonary fibrosis than did those with anti-RNAP (Table 2). These results suggest that the pathogenesis of SSc in patients with anti-topo I might be mediated by more severe vascular damage compared with those with anti-RNAP. Another striking finding was the incidence of renal involvement, termed renal crisis. Since the occurrence rate of renal crisis is strongly associated with anti-RNAP,^9^,^34^ the presence of anti-RNAP can guide clinicians to careful follow-up for the incidence of this complication. Our results showed that all three patients with renal crisis had anti-topo I with no incidence in 12 anti-RNAP-positive patients (Tables 2 and 3). We currently do not have enough data to account for this discrepancy from previous reports. It may be due to the difference in genetic background, as the incidence of renal crisis is generally low in Japanese patients with SSc. Nonetheless, a previous report demonstrated that 43% of patients with anti-RNAP had renal crisis and the presence of anti-RNAP was associated with both SSc-related and all-cause mortality in Japanese patients with SSc.^30^ As the previous report was analysed more than 10 years ago, a possible explanation is the change of environment or treatment during the period. Alternatively, this difference may be explained by the fact that our study was conducted in dermatological clinics. Further large, multicentre longitudinal studies are required to assess the incidence of renal crisis and the association of renal crisis with ANA in Japanese patients with SSc.

We confirmed that most patients with ACA are female and have lSSc, longer disease duration and infrequent internal organ involvement, except for oesophageal dysfunction. However, frequencies of vascular damage are different between Japanese and Caucasian and/or black African patients. While frequencies of pitting scars or ulcers were 42–61% in Caucasian and/or black African subjects,^29^,^35^,^36^ our data showed that only 17% of ACA-positive patients had pitting scars (Table 1). Steen reported that isolated PAH was detected in 19% of patients with ACA leading to a decreased survival rate.^29^ In contrast, only 7% of ACA-positive patients in our study showed isolated PAH and it was not severe except in one patient who died (Tables 1 and 3, and data not shown). Thus, the clinical presentation of ACA-positive patients is generally similar among different ethnic groups, except for the lower risk or lower severity of vascular involvement in Japanese patients.

Patients with anti-Th/To have been reported to have more subtle cutaneous, vascular and gastrointestinal involvement, but more often have symptoms commonly seen in dSSc, such as pulmonary fibrosis and isolated PAH, as well as reduced survival compared with patients with ACA.^15^,^29^,^35^ In contrast, internal organ involvement except for oesophageal dysfunction is infrequently seen in Japanese patients with anti-Th/To.^30^ In this study, patients with anti-Th/To had lSSc, with few complications from organ involvement (Table 1). Also, previous studies have shown that patients with anti-U3 RNP have severe cutaneous and internal organ involvement.^29^,^37^,^38^ On the other hand, lower frequencies and less severe internal organ involvement were described in Japanese patients with anti-U3 RNP.^30^ In our study, patients with anti-U3 RNP had dSSc but infrequent internal organ involvement except for oesophageal dysfunction, similar to those with anti-Th/To. It may be reasonable to conclude that Japanese patients with the ANA, anti-Th/To and anti-U3 RNP, have infrequent or milder internal organ involvement with better survival compared with Caucasian and/or black African patients with these ANA.

Little has been documented concerning the clinical characteristics of ANA-negative SSc patients. In our study, they had relatively short disease duration and infrequently had vascular and cutaneous involvement. Pulmonary fibrosis and isolated PAH rarely occurred, but a decrease in %DLco was commonly seen. Oesophageal dysfunction occurred in 60% of this subgroup; however, heart and renal involvement rarely occurred. Together, these results suggest that patients with SSc without ANA have infrequent or milder organ involvement. However, it is noteworthy that one patient in this subgroup died of continuous bleeding in both the upper and lower gastrointestinal tracts related to SSc. Therefore, it is important to keep in mind that the absence of ANA in patients with SSc may not be an indicative marker for specific clinical feature but represents the heterogeneity of the population.

It has been reported that patients with anti-RNAP have the worst survival rate.^29^,^30^ However, mortality in patients with anti-RNAP is currently lower than in those with anti-topo I.^29^ This is because patients with anti-RNAP have a low risk of suffering pulmonary fibrosis, and renal crisis is now more readily treated with angiotensin-converting enzyme inhibitors than pulmonary fibrosis.^39^ Our data indicate that patients with anti-topo I have the worst survival rate of all subgroups and that the prognosis of other patients with SSc is favourable (Fig. 2b). Thus, anti-topo I is currently probably the only marker for poor prognosis in Japanese patients.

In conclusion, grouping patients with SSc according to their serum ANA may guide the clinician to focus on particular risks during follow-up in individual patients. However, we should take into account the ethnic and genetic background as several clinical features in each SSc-related ANA-based subgroup appear to vary among populations of different backgrounds. Moreover, since the number of Japanese patients reported with anti-RNAP, anti-Th/To or anti-U3 RNP is small, more studies are essential for the better understanding of Japanese patients with SSc with these ANA.
